# Prevalence of Gestational Diabetes Mellitus, Postpartum Depression, and Hypertension Affecting Pregnancies in Rural Ohio

**DOI:** 10.1089/whr.2024.0137

**Published:** 2025-01-28

**Authors:** Amber M. Healy, Mallory Faherty, Jordan Ackerman, Blake Leeds, Mandy Morell, Rachel Kim, Andrew Ackerman, Jody M. Gerome

**Affiliations:** ^1^ Ohio University, Heritage College of Osteopathic Medicine, Athens, Ohio, USA.; ^2^ Ohio Health Physician Group, Heritage College Diabetes and Endocrinology, Athens, Ohio, USA.; ^3^ Ohio Health Research Institute, Columbus, Ohio, USA.; ^4^ Aultman Hospital, Canton, Ohio, USA.; ^5^ Ohio Health Doctors Hospital, Columbus, Ohio, USA.; ^6^ Cleveland Clinic Akron General, Akron, Ohio, USA.; ^7^ Ohio Health Physician Group, Heritage College Obstetrics and Gynecology, Athens, Ohio, USA.

**Keywords:** gestational diabetes, hypertension in pregnancy, postpartum depression

## Abstract

**Objective:**

Determine if diagnosis of gestational diabetes mellitus (GDM) increases the incidence of postpartum depression (PPD) in rural Appalachian Ohio.

**Methods:**

A retrospective chart review was conducted to review pregnancies between February 2020 and March 2023 to look for diagnoses of gestational diabetes and PPD.

**Results:**

The population studied showed that 5.41% of pregnancies were affected by gestational diabetes, 7.76% of pregnancies had associated PPD, and 1.26% had both. Hypertension incidence that was a secondary data point in this study showed that 20.4% of pregnancies were affected by a form of hypertension.

**Conclusions:**

Incidence of gestational diabetes and PPD were similar in this population to the rest of the United States and occurred together at low rates. Hypertension was a more common condition that affected pregnancies in this population.

## Introduction

Gestational diabetes mellitus (GDM) is defined as an intolerance to carbohydrates that develops during pregnancy.^1^ Approximately 7% of pregnancies are affected by some form of diabetes mellitus, with the majority categorized as GDM.^1^ Heightened rates of GDM have been documented over the past few decades, stemming from predisposing psychological, social, and genetic risk factors.^2–4^ Diabetes in Appalachian Ohio has been shown to have a higher prevalence than other parts of the United States with estimations of 1 in 5 people affected by diabetes compared with 1 in 10, respectively.^5,6^ Furthermore, it is estimated that two-thirds of the population in the region are categorized as being overweight or having obesity.^5^

Another potential complication of pregnancy, postpartum depression (PPD), affects 15%–20% of women who successfully give birth.^7,8^ The effects of PPD can negatively influence the health behaviors of women and their children.^9^ PPD is defined as mood changes in a major depressive episode that occurs within 4 weeks after delivery.^10^ Risk factors for PPD include anxiety or depression during pregnancy, stressors during pregnancy or the early postpartum period, traumatic birth experiences, preterm birth, admission of infant to neonatal intensive care, problems breastfeeding, lack of social support, and history of depression.^11^

The prevalence of depression is twice that of the general population in patients with type 1 or 2 diabetes.^12^ This association has solidified the understanding that phenotypes for depression and glucose intolerance have a bidirectional relationship and are exceedingly comorbid.^13^ A prospective study by Hinkle et al. demonstrated a slight increase in incidence of GDM for patients who developed depressive symptoms early during their pregnancy.^14^ Furthermore, this study found a modest association between GDM and developing PPD.^14^ A large meta-analysis found a 32% increase in the risk of developing PPD in patients who had developed GDM. This meta-analysis also suggested that retrospective studies of the correlation between GDM and PPD contained lower estimates than prospective studies.^15^ Proposed mechanisms for how PPD can develop in the presence of GDM are not well understood, but theories include a role of insulin resistance, elevated proinflammatory markers, and/or dysregulation of the hypothalamic–pituitary–adrenal axis.^16^ Contrary to these studies, a systematic review by Ross et al. declared that there was no clear correlation between GDM and developing depression during pregnancy or in the postpartum period.^17^ Given the results of these analyses, the relationship between GDM and PPD is still not widely understood.

Appalachian Ohio is a rural region impacted by poverty and poor access to mental health resources, both of which impact social drivers of health. Poor nutrition and elevated cortisol levels from stress are factors that are more prevalent in people experiencing poverty and are known to affect health.^18^ In Appalachian Ohio, the rate of people living in poverty is 27% greater than that of the United States.^19,20^ Yearly median income in Appalachian Ohio is 28% less than the US median income.^19,21^ by households in Appalachian Ohio at a 16% higher rate compared to households in the state of Ohio or the United States.^19,22^ In this region, 5%–8.6% of the population lacks health insurance, which is similar to the United States.^19,23^ There are 20.6% of Appalachian Ohio adults enrolled in Medicaid.^24^ In Ohio, eligibility for Medicaid coverage in pregnant women begins at 200% of the federal poverty level, which is dependent on household size.^25^ Mental health resources are typically lacking in rural areas and counties, and Appalachian Ohio is no different. It is identified as a high Mental Health Professional Shortage Area.^26^ This study looked at rates of gestational diabetes, PPD, and rates of having both gestational diabetes and PPD in a rural Appalachian region. With the higher rates of obesity, we suspect higher rates of GDM. Similarly, with higher rates of poverty and its impact on stress levels, we suspect higher rates of GDM and PPD.

## Methods

This study was deemed exempt by the institutional review board and data were collected and stored in REDCap (Research Electronic Data Capture). A retrospective chart review of a single center was conducted for patients and their pregnancies delivered between February 24, 2020, and March 31, 2023. Patient charts were identified using International Classification of Diseases, 10th Revision code O24.XXX who received care in Southeast Ohio during their pregnancies. The retrospective chart review was completed by a group of physicians who reviewed the criteria for consistency of documentation.

Patient charts were included for all live births after 37 weeks gestation. Each pregnancy was considered as a separate episode. Patients with multiple episodes were included for each individually if inclusion criteria were met. Charts were excluded if the patient had a previous diagnosis of depression or PPD in the last 5 years, a preexisting history of type 1 or 2 diabetes, diagnosis of substance misuse disorder social history, documentation of alcohol abuse, illicit substance use including marijuana, or medication-assisted therapy for substance abuse.

After meeting initial inclusion criteria, the charts were further reviewed. Absent documentation for a given variable was left blank. Demographic data were collected including patient age, race, marital status, and insurance type. Insurance type was classified as commercial or government-issued (Medicaid and Medicare plans). Other descriptive information about gestational age at delivery and smoking status were also recorded. Data recorded for the diagnosis of gestational diabetes was determined by glucose tolerance testing and use of the Carpenter–Coustan criteria as described below. Data regarding the diagnosis of PPD was determined by utilizing the Edinburgh Postnatal Depression Screen (EPDS). With respect to criteria for gestational diabetes, the patient had to complete a two-step oral glucose tolerance test where the patient had the 50-g glucose load with 1 hour glucose over 135 mg/dL and then followed up with the 100-g glucose load with 3 hours of glucose readings where two of the four readings were considered abnormal in pregnancy. Those abnormal thresholds were defined as greater than 95 mg/dL fasting, greater than 180 mg/dL after 1 hour, greater than 155 mg/dL after 2 hours, and greater than 140 mg/dL after 3 hours.^27,28^ The exception to this was patients who had blood sugar readings over 200 mg/dL after the 1 hour, 50-g test as they met criteria of GDM diagnosis per guidelines. Any patient with a score of 10 or greater on the EPDS was determined to have PPD.

Secondary endpoints included previous GDM, GDM diagnosis in the previous 5 years and incidence of hypertension affecting pregnancy, including preexisting hypertension, pregnancy-induced hypertension, gestational hypertension, and preeclampsia all coded under hypertension.

All statistical analysis was performed utilizing JASP 0.17.1.0 and Jamovi 2.3.21.0. Descriptive statistics were calculated to describe and summarize the data. Means and standard deviations were utilized to describe continuous variables. Frequencies and percentages were utilized to describe categorical variables. Differences between groups were calculated utilizing analysis of variances (ANOVAs) and chi-squared tests. The *p*-value was set at ≤0.05 *a priori*.

Charts with missing variables were included in the data analysis because the nature of the initial collection of this data, patient care encounters, often leads to missing data. Excluding patients, variables, or outcomes based on missing data may have skewed our results. The statistical analyses were run with the number of variables present so the *n* of each variable may not be equal to the number of charts that met inclusion criteria for this review.

## Results

There were 554 pregnancies included in this chart reviewed after screening each for inclusion and exclusion criteria. The average age of pregnant women was 28.39 ± 5.59 years. The average gestational age at delivery was 38.55 weeks and 2.39 days. The characteristics of the pregnancies are included in Table 1.

**Table 1.** The Demographics, Insurance Type, and Smoking Status of Charts Included for Review in This Study

	% (*n*)
Race/ethnicity	
Caucasian	90.43% (501)
African American	3.79% (21)
Asian	1.99% (11)
Hispanic	0.36% (2)
Native American/Alaskan	19% (1)
Latin American	0% (0)
Pacific Islander/Hawaiian	0% (0)
Not available	3.25% (18)
Marital status	
Single	23.83% (132)
Married	73.83% (409)
Separated	0.36% (2)
Divorced	0.90%(5)
Widow	0% (0)
Not available	10.83% (6)
Insurance type	
Commercial	56.14% (311)
Government	39.35%(218)
Not available	4.51%(25)
Smoking status	
Nonsmoker	85.92% (476)
Former smoker	11.55% (64)
Current Smoker	1.99% (11)
Not available	0.54% (3)

Primary endpoints showed that 5.41% (*n* = 30) pregnancies were affected by gestational diabetes, 7.76% (*n* = 43) pregnancies were affected by PPD. There were 1.26% (*n* = 7) pregnancies identified as affected by both gestational diabetes and PPD. Further details about these different patient groups are included in Table 2.

**Table 2.** Incidence of Postpartum Depression and Gestational Diabetes in Different Demographics, Insurance Types, Smoking Status, and Incidence of Comorbid Hypertension Affecting Pregnancies

	PPD *n* = 43	GDM*n* = 30	PPD and GDM*n* = 7
Race/ethnicity			
White	86.05% (37)	86.67% (26)	85.70% (6)
African American	2.33% (1)	3.33% (1)	0% (0)
Asian	0% (0)	6.67% (2)	14.29% (1)
Hispanic	0% (0)	0% (0)	0% (0)
Native American/Alaskan	2.33% (1)	0% (0)	0% (0)
Latin American	0% (0)	0% (0)	0% (0)
Pacific Islander/Hawaiian	0% (0)	0% (0)	0% (0)
Not available	9.30% (4)	3.33% (1)	0% (0)
Marital status			
Single	25.58% (11)	80.00% (24)	14.29% (1)
Married	72.09% (31)	20.005 (6)	85.70% (6)
Separated	0% (0)	0% (0)	0% (0)
Divorced	2.33% (1)	0% (0)	0% (0)
Widow	0% (0)	0% (0)	0% (0)
Insurance type			
Commercial	48.84% (21)	63.33% (19)	42.86% (3)
Government	48.64% (21)	36.67% (11)	42.86% (3)
Not available	2.33% (1)	0% (0)	14.29% (1)
Smoking status			
Nonsmoker	79.07% (34)	86.67% (26)	100.00% (7)
Former smoker	16.28% (7)	10.00% (3)	0% (0)
Current smoker	4.65% (2)	3.33% (1)	0% (0)
Hypertension	23.26% (10)	36.67% (11)	42.86% (3)

GDM, gestational diabetes mellitus; PPD, postpartum depression.

Secondary endpoints showed that there were 20.40% (*n* = 113) pregnancies affected by hypertension. Few women had a previous pregnancy affected by gestational diabetes in the previous 5 years (*n* = 24) or otherwise (*n* = 20).

Further statistical analysis of the relationship between GDM and PPD. Chi-squared test and ANOVA analysis of age, gestational age at delivery, race, insurance type, marital status, smoking status, and presence of the comorbid condition of hypertension (HTN). Chi-squared test analysis and ANOVA results are shown in Table 3. The original aim of the study to look at GDM as a risk factor for development of PPD in the study population was not statistically significant with either ANOVA or chi-squared test (see Table 4).

**Table 3. This Shows that There was not Statistical Significance for Demographics, Smoking Status, or Co-morbidity of HTN in Pregnancy Influencing GDM and PPD in the Study Population:** 

	GDM and PPD		
	ANOVA	Chi-squared test	*p*
Age	1.967		0.147
Gestational age	1.119		0.332
Race ethnicity		5.279	0.948
Marital status		3.976	0.859
Insurance type		1.326	0.515
Smoking status		2.249	0.690
HTN		2.117	0.347

ANOVA, analysis of variance; HTN, hypertension.

**Table 4.** Gestational Diabetes as a Risk Factor for the Development of Postpartum Depression Was Not Statistically Significant in This Study Population

	Incidence of PPD	Chi-squared test	*p*
Positive screen for GDM			
Yes (*n* = 37)	7 (18.92%)	2.918	0.088
No (*n* = 434)	43 (9.91%)		

## Discussion

Our study population had a rate of gestational diabetes of 5.41%, which is comparable to that of the Centers for Disease Control and Prevention (CDC)-reported average of 5%–9% of affected pregnancies each year.^29^ The rate of PPD in our study population was found to be 7.76%, which is less than the 15%–20% previously reported. Both the rates of GDM and PPD were lower than expected based on the typical frequency in the region studied. Diabetes rates in the southeastern Ohio region and rates of depression in Ohio are higher than the national average. Of note, the rates of elevated blood pressure affecting pregnancy appeared to be higher (20.4%). The CDC reports that 6%–8% of pregnancies are affected by high blood pressure nationally.^30^

While we recognize that diabetes and depression are both classified as chronic disease states, we expected that their rates could inform our study and its focus on the more acute variants, GDM and PPD. Rates of both diabetes and depression in Ohio’s population have recently increased, notably at younger ages. One report revealed that depression rates are higher in women than in men, and in all individuals, depression rates are greater under the age of 45 in comparison to older adults.^31^ Characteristics of the population included could be confounding factors. Our sample included individuals from the Appalachian region. Community is a central part of Appalachian culture, including strong family and church support through faith. People of this region tend to approach family members or religious leaders when they require assistance with personal, family, or health concerns.^32^ They also tend to be protective of their family and suspicious of outsiders which leads to a decreased willingness to share the problems that they experience with health professionals.^32^ A strong familial and community support structure is a factor that was not considered in this study that could have led to a lower rate of PPD. The data set also includes individuals who are connected to a large state-supported university. The university population has proven to be an outlier in the previous regional needs assessment, demonstrating a lower prevalence of diabetes than the county where it is located.^5^

Our inclusion criteria were strict. We excluded depression, substance abuse disorder, impaired glucose tolerance, elevated hemoglobin A1c, and preexisting diabetes from this chart review. By using elevated hemoglobin A1c, used in first-trimester screening, also decreased the number of second-trimester screenings. If those individuals had been included the numbers for both PPD and GDM would have been higher than the average in our population. A future research consideration should be looking at all categories of glucose abnormalities in pregnancy since prediabetes is underdiagnosed in women of childbearing age.^33^ Further consideration of mental health concerns in pregnancy and their prevalence in Appalachian Ohio could also warrant investigation.

Discovery of the higher rates of HTN affecting pregnancy was an unexpected finding. Obesity, smoking, alcohol use, sedentary lifestyle, unhealthy diets, and genetics are risk factors for HTN.^34^ Although weight and body mass index metrics were not collected, it was previously reported that two-thirds of the population in our region reported being overweight or having obesity.^5^ Obesity is likely a reason for higher HTN rates in our study population. Smoking is a risk factor for HTN, but our study population was mostly never smokers, so this is not likely a contributing factor to our finding. Alcohol abuse was an exclusion criterion so is not a factor captured in our study population. Information about lifestyle habits with respect to physical activity level and diet was not available to determine how our study population is impacted. A future prospective study looking at HTN risk factors and their impact on pregnancy would help to answer some of these questions.

## Limitations

Like many retrospective chart reviews, this study design had limitations. Despite the use of a common electronic medical record (EMR), we anticipate there were inaccurate and incomplete data and variance in data quality. Although it is likely that healthcare providers documented medical diagnoses differently, there was a consistent process for completing and documenting the EPDS. Patient refusal to complete the entire protocol for GDM diagnosis led to exclusion from the review due to the lack of completion of the diagnostic test, although these individuals did receive care as suspected GDM. Notably, the period of this chart review coincided with the introduction of a new EMR, which may have impacted the data recorded in the patient medical record, especially with completion for the EPDS. We noticed that the EPDS was more consistently completed in patients when the data were collected more remotely from the EMR launch date.

Another limiting factor in the data collection was a way to determine financial stability. There is not a uniform way that social drivers of health (SDOH) data are collected in the EMR, but the most consistent marker was insurance type. Most patients reviewed who had government insurance were Medicaid beneficiaries. Future prospective studies where patients report their SDOH data, and it is correlation with GDM- and PPD-rated would be beneficial.

## Conclusion

Diagnosis of gestational diabetes does not appear to increase the risk of having PPD in our study population in southeastern Ohio. Surprisingly, despite the high rates of diabetes in the region, the rate of gestational diabetes was comparable to the national data. Notably, the rates of all types of hypertension disorders in pregnancy were greater in our population.

## Abbreviations Used

CDC: Centers for Disease Control and Prevention
EMR: electronic medical record
EPDS: Edinburgh Postnatal Depression Screen
GDM: gestational diabetes
HTN: hypertension
PPD: postpartum depression
SDOH: social drivers of health
